# Deep learning for motion classification in ankle exoskeletons using surface EMG and IMU signals

**DOI:** 10.1038/s41598-025-22103-1

**Published:** 2025-10-31

**Authors:** Silas Ruhrberg Estévez, Josée Mallah, Dominika Kazieczko, Chenyu Tang, Luigi G. Occhipinti

**Affiliations:** 1 Electrical Engineering Division, Department of Engineering, University of Cambridge, Cambridge, CB3 0FA UK; 2 School of Clinical Medicine, University of Cambridge, Cambridge, CB2 0SP UK

**Keywords:** EMG, IMU, Exoskeleton, Ankle, Machine learning, Biomedical engineering, Electronic properties and devices

## Abstract

Ankle exoskeletons have garnered considerable interest for their potential to enhance mobility, support rehabilitation, and reduce fall risks, particularly among the aging population. Their effectiveness depends on accurate, real-time prediction of user intentions from wearable sensor data, as even small delays or errors can compromise stability and safety. Here, we present a motion classification framework that integrates three Inertial Measurement Units (IMUs) with eight surface Electromyography (sEMG) sensors fabricated as towel-based textile electrodes, which improve comfort, durability, and usability for long-term deployment compared to traditional gel electrodes. The dataset comprises multichannel time-series recordings of five functional daily motions, enabling a realistic evaluation of exoskeleton use in everyday environments. Using this framework, Convolutional Neural Networks (CNNs) achieved an accuracy of $99.263 \pm 0.26\%$, substantially surpassing previously reported results in the field. Beyond overall accuracy, we address deployment-critical requirements: transfer learning enables reliable adaptation to new users with as few as ten calibration samples per motion, while robustness testing demonstrates that the system continues to provide stable classification even when individual sensors are disrupted. Together, these results highlight the feasibility of safe, high-accuracy, and real-world-ready exoskeleton control through deep learning combined with wearable textile electrodes and IMUs.

## Introduction

Globally, approximately one in ten people is over the age of 65, a proportion that is expected to double by the end of the century^1^. Among the elderly, an estimated $32\%$ experience gait disturbances, which significantly increase their risk of falls^2^. In the United States and the United Kingdom, the annual cost of falls is estimated at $\$50$ billion^3^ and $\$2.6$ billion^4^, respectively. Ankle exoskeletons offer a promising solution to counteract the decline in muscle strength and balance associated with aging, potentially improving gait and reducing the risk of falls^5,6^. Additionally, these devices may benefit patients suffering from conditions such as stroke, multiple sclerosis (MS), and cerebral palsy^7–9^, while also supporting healthy individuals during physically demanding work or to reduce fatigue^10–14^.

Exoskeletons can be broadly categorized as either active or passive, based on the presence of an external power source^15^. Passive exoskeletons rely on mechanical components, such as springs, to assist walking by storing and releasing energy at specific points in the gait cycle^16^. In contrast, active exoskeletons incorporate actuators like electric motors or pneumatic systems to directly reduce the physical effort required for walking, and in some cases, enable walking without muscle activity^17,18^. For optimal support, active exoskeletons must accurately classify the user’s motion intentions and minimize latency between sensor input and control response^19^. Common sensors used in lower limb applications include inertial measurement units (IMUs), surface electromyography (sEMG), and force or pressure sensors^20,21^.

Machine learning techniques such as Support Vector Machines (SVM), Convolutional Neural Networks (CNN), and Long Short-Term Memory (LSTM) networks have been widely applied to this task. While SVMs are simple and effective for small datasets, they scale poorly to high-dimensional multi-class problems and are not well suited to deployment in real-time wearable systems^22^. Deep learning approaches, by contrast, can jointly learn representations from multimodal signals and adapt across users. Prior work has demonstrated encouraging but limited results: Li et al. achieved 95% accuracy on isolated ankle movements using CNNs and LSTMs^23^, Cheng et al. reported similar findings with a CNN–LSTM hybrid^24^, and Kim et al. achieved 88% accuracy when classifying daily living activities from EMG and IMU data^25^. While promising, these accuracies remain insufficient for seamless and safe exoskeleton operation, since even modest misclassification rates in lower-limb devices can increase the risk of instability and injury^20^.

In this study, we evaluate whether scalable models such as LSTM and CNN can achieve robust, real-world motion classification using non-invasive EMG and IMU recordings. Our dataset consists of multichannel time-series signals from five representative daily motions, enabling realistic assessment beyond isolated ankle movements. Our contributions are fourfold. First, we demonstrate that CNNs achieve accuracies above 99% on these time-series recordings, substantially surpassing previous studies. Second, we introduce towel-based textile electrodes that improve usability and comfort for long-term monitoring, while maintaining high signal quality^26^. Third, we assess deployment-critical scenarios, showing that performance generalizes across new users with transfer learning and remains well above 80% even when individual sensors fail. Finally, we release our dataset, code, and models to support the development of next-generation classification methods for safe and practical exoskeleton control.

## Results

### Data collection from human subjects

**Fig. 1:**
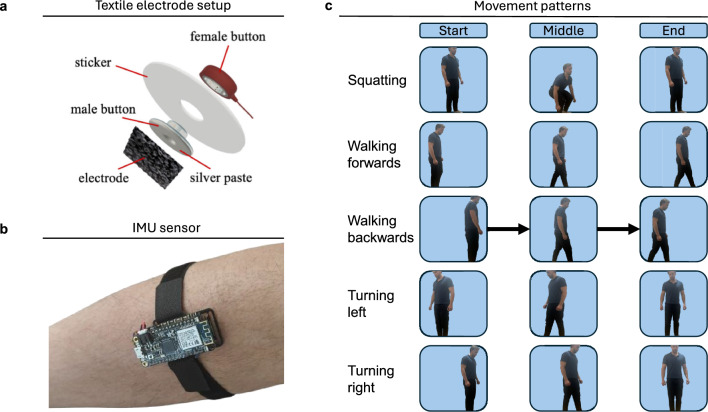
Data collection. (**a**) Schematic of the custom-made textile electrode assembly used for EMG recordings^26^. (**b**) Motion data acquisition using commercial IMUs secured with elastic straps. (**c**) Overview of the recorded activities: squatting, walking forwards, walking backwards, and turning left or right. Representative examples of each activity are shown, illustrating the start, intermediate, and end positions.

Ethical approval was obtained to record a total of 1,504 trials across three subjects using both EMG and IMU sensors (see Fig. 1). IMU sensors were positioned on both shanks and on the right foot, while surface EMG sensors were placed to capture activity of the tibialis anterior, gastrocnemius medial and lateral heads, and soleus muscles. The five recorded motions, chosen as essential for navigating a barrier-free environment, included walking forwards, walking backwards, turning left, turning right, and squatting (to pick up an object or sit down). All recordings consisted of multichannel time-series signals, which were pre-processed and subsequently used to train the motion classifier.

**Fig. 2:**
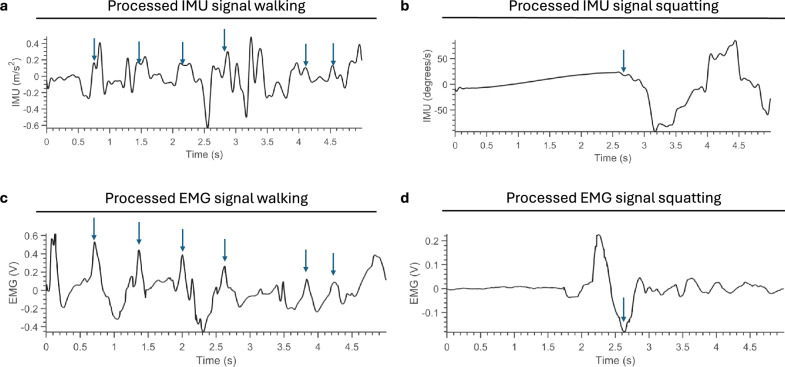
Signal recordings. Representative examples of the relationship between recorded signals and physiological processes (**a**-**d**). During forward walking, intermittent forward acceleration is detected by the IMU (**a**), which corresponds to regular activation of the soleus muscle (**c**). Similarly, during a squat movement, slight forward motion of the shank is observed by the IMUs, which is linked to activity in the tibialis anterior muscle (**d**). Blue arrows highlight key instances where the two signals align, emphasizing representative matches. The EMG signal leads the IMU data, as there is an inherent latency between the electrical signals indicating muscle contraction and the subsequent initiation of motion detected by the IMU.

Representative raw recordings from two trials are shown in Fig. 2, illustrating characteristic patterns linking muscular activation to kinematic signals. During forward walking, the IMU’s vertical acceleration exhibits distinct peaks corresponding to individual steps, although some strides lack clear peaks when the foot is not lifted significantly. In parallel, the soleus EMG shows multiple activations, consistent with its role in plantarflexion during propulsion^27^. In contrast, the squatting motion is characterized by activation of the tibialis anterior, required for dorsiflexion to advance the knees forwards^28^, followed by a marked increase in anteroposterior acceleration. These examples demonstrate the physiological plausibility of the recorded time-series and their suitability for motion classification.

### Motion classification using deep learning

**Fig. 3:**
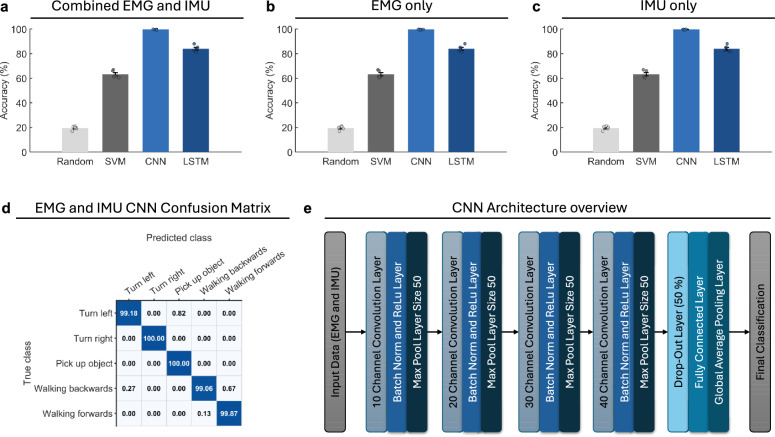
Classification accuracy of different models. (**a**) Performance of CNN, LSTM, and SVM models using combined EMG and IMU data. (**b**) Model performance using EMG data only. (**c**) Model performance using IMU data only. (**d**) Confusion matrix illustrating per-class performance of the CNN. (**e**) Overview of the CNN architecture used in this study.

For classification, both LSTM and CNN models were trained on IMU data, EMG data, and the combined IMU and EMG data. Combining IMU and EMG data for both models resulted in higher performance (see Fig. 3). The CNN demonstrated a clear performance advantage over the LSTM, achieving an accuracy of $99.263 \pm 0.26\%$ on the test data, compared to the LSTM’s $83.96 \pm 2.64\%$ and the SVM baseline of $62.66 \pm 3.28\%$. Models trained on individual modalities, such as EMG or IMU data alone showed similar performance (see Fig.e 3). Additionally, given that some users may require exoskeleton support for only one leg, we evaluated classification accuracy using EMG and IMU data from a single leg. In this case, the CNN achieved accuracies remaining above $99\%$ for both the leg instrumented with two IMU sensors and the leg instrumented with one IMU sensor, demonstrating that robust classification is maintained even under reduced sensor availability, a scenario highly relevant to real-world exoskeleton use in patients recovering from strokes and other unilateral neurological deficits^29^.

A detailed performance analysis of the CNN on test data using all channels is shown using a confusion matrix (Fig. 3) and precision, recall, and F1 scores for the five classes (Table 1). The CNN demonstrates high accuracy across all classes, with only minor confusion observed between certain motions. Specifically, slight misclassification occurred between Walking Forwards and Walking Backwards, which is likely attributable to the engagement of overlapping muscle groups and the symmetrical nature of leg movements, despite their opposing directions^30^. Importantly, overall classification accuracy remained consistently high across all classes.

**Table 1:** Summary metrics (Accuracy, Precision, Recall, F1) of CNN motion classification performance using all channels, reported as mean ± standard deviation across $n=5$ runs.

Metric	Value
Accuracy	$0.9963 \pm 0.0026$
Precision (macro)	$0.9964 \pm 0.0025$
Recall (macro)	$0.9962 \pm 0.0025$
F1 (macro)	$0.9963 \pm 0.0025$

### Transfer learning for model deployment

**Fig. 4:**
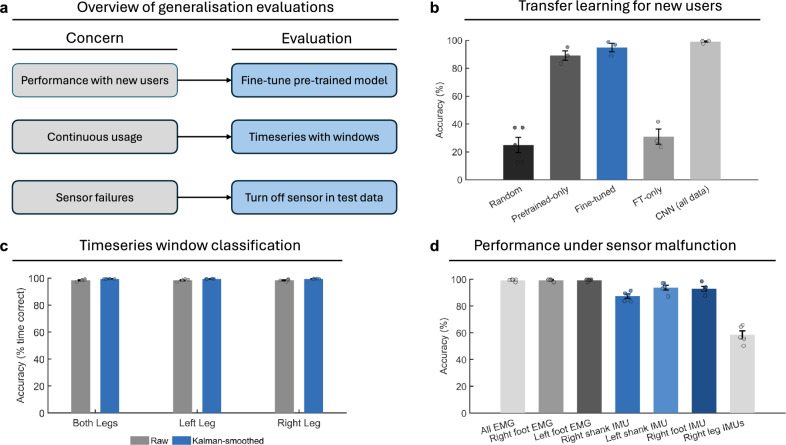
Transfer learning and model robustness. (**a**) Key challenges for exoskeleton deployment and the corresponding evaluations performed to assess model generalizability. (**b**) Pre-trained models exhibit strong cross-subject generalization, with performance further improved through fine-tuning on a small calibration set. (**c**) In the continuous classification setting, systems using data from either a single foot or both feet achieve high accuracy, which is further enhanced by applying Kalman smoothing to the predictions. (**d**) Impact of simulated sensor failures on classification accuracy, illustrating the relative importance of individual sensors and the overall robustness of the framework.

To evaluate cross-user generalization, we adopted a leave-one-subject-out protocol in which each of the three subjects was used once as the held-out target, while the CNN was trained on data from the remaining two subjects. For the target subject, the model was first pre-trained on the source subjects and then adapted using a small calibration subset from the target: 10 samples per class, with performance evaluated on the remaining samples of that subject. Averaged across all three subjects, the pre-trained-only CNN achieved $89.21 \pm 5.88\%$ accuracy without any calibration. When trained from scratch on the few-shot calibration subset alone (FT-only, no pre-training), performance dropped markedly to $31.04 \pm 9.48\%$. In contrast, fine-tuning the pre-trained model with the small calibration set improved accuracy to $94.90 \pm 5.28\%$. For fine-tuning, the full network was updated at a reduced learning rate while freezing batch-normalization statistics, which stabilized adaptation to the target subject under few-shot conditions. These results demonstrate that transfer learning substantially improves generalization to unseen users, enabling reliable classification even when only a handful of calibration samples are available (see Fig. 4).

### Time-series window classification

In real-world deployment, the model must dynamically predict user intent from continuous data streams rather than isolated motion segments. To approximate this scenario, we generated a continuous dataset by randomly concatenating all five motion classes and then applied the trained CNNs using signals from both legs as well as from each leg individually. A sliding window of 5 s was moved across the data with strides of 1 s or 2 s, and each segment was classified. Without any post-processing, the CNN achieved an accuracy of 98.42(39)% with a stride of 1 s and 98.53(36)% with a stride of 2s. Applying a Kalman filter to smooth the predictions further improved performance to 99.41(11)% and 98.98(5)%, respectively. Comparable results were obtained when using sensors from the left or right leg individually, confirming that robust continuous classification is possible even under reduced sensor configurations (see Fig. 4).

### Model robustness to sensor failure during operation

During real-world use, individual sensors may fail to provide reliable signals. For example, EMG electrodes can lose contact with the skin, or an IMU battery may deplete. In such cases, model performance is expected to decrease due to the missing inputs. However, it is essential that classification remains sufficiently accurate to ensure safe exoskeleton operation, allowing the user to retain mobility until the faulty sensor can be replaced. To evaluate this scenario, we simulated sensor failures by setting all channels from a given sensor to zero in the test set, while leaving training data unchanged.

The CNN maintained high classification accuracy when EMG electrodes from either leg were removed, with performance remaining at $99.20 \pm 0.87 \%$. In contrast, the system was more sensitive to the loss of IMU signals. Removing the right shank IMU reduced accuracy to $87.24 \pm 3.54\%$, while removing the left shank IMU yielded $93.69 \pm 4.10\%$. Loss of the right foot IMU decreased accuracy further to $92.89 \pm 3.85\%$. The largest degradation occurred when both IMUs on the right leg were removed, resulting in a drop to $58.47 \pm 6.54\%$. These findings highlight that while the model is generally robust to individual sensor failures, the right leg IMUs, particularly when considered together, provide critical information for reliable classification (see Fig. 4). Although models trained on signals from individual legs achieve accuracies above 99 %, in the failure scenario the loss of a single shank IMU appears to be partially compensated by the remaining sensors, whereas simultaneous loss of both right leg IMUs cannot be recovered in this way.

## Discussion

We demonstrate that EMG and IMU signals can be effectively utilized for high-accuracy motion intention prediction using a CNN. The proposed prototype is cost-efficient, built with commercially available IMU sensors and EMG electrodes, with minor modifications to the contact pads. Additionally, the machine learning models are lightweight, eliminating the need for external graphics cards during operation. Our model outperforms comparable models for lower limb motion classification reported in the literature (see Table 2). Previous work has shown that high accuracies can be achieved using traditional machine learning techniques for ankle motion classifications^31–33^. However, in recent years deep learning techniques such as CNNs have shown higher performance on many tasks due to the ability to train on large amounts of data and task specific fine-tuning^34^. We also demonstrate limitations of traditional baselines like SVMs when dealing with high-dimensional multi-class data.

**Table 2:** Comparison of classification performance with similar machine learning models reported in the literature.

Study	Classes	Movements	Sensors	Model	Accuracy
Kim et al.	6	Full body	IMU + EMG	CNN	88.0%
Li et al.	6	Ankle only	EMG	CNN–LSTM	95.7%
Si et al.	5	Full body	EMG	CNN	95.5%
This work	5	Full body	IMU + EMG	CNN	**99.2%**

Importantly, unlike the work by Li et al., our study involved full-body movements rather than limiting classification to isolated ankle motions, which are typically required for exoskeleton support^23^. This distinction makes direct comparisons challenging, as the movement classes we trained for are substantially different. The most comparable model in terms of sensor setup is that of Kim et al., which also focused on motions needed for navigating an environment but achieved only 88% accuracy^25^. Additionally, the model by Si et al. achieved accuracy closer to ours using only EMG recordings, but it focused on movements not commonly performed in daily life, such as a straight leg lift, which involve very different muscle activation patterns which likely facilitate classification^35^.

The accuracy of our CNN trained solely on the EMG portion of the dataset is lower than those of the studies that performed isolated ankle muscles. This is likely due to muscle signal cross-talk when performing complex movements^36^. Notably, the model also demonstrated strong performance when trained using only the IMU data. This is particularly promising, as muscle atrophy in paralyzed patients can lead to altered or diminished EMG signals, making IMU data a valuable alternative^37^. These findings underscore that while each modality can be effective for motion classification on its own, sensor fusion should be prioritized for more accurate and robust system development.

To the best of our knowledge, this is the first approach to incorporate textile electrodes in wearable exoskeleton control systems, which offer greater promise for long-term monitoring due to their comfort and wearability. Unlike previous studies, we specifically focused on evaluating model performance for motions that are most critical to exoskeleton operation. Furthermore, we conducted additional analyses to assess the feasibility of deploying our model in real-world settings, as well as its safety for users, ensuring that the system is both practical and secure for continuous use in assistive devices. We show that our model maintains very high accuracy when developed in a simulated real-world continuous classification setting.

To effectively deploy an exoskeleton to new users, it is essential that the model generalizes well beyond the initial training dataset. Retraining the model for each individual would be both time-consuming and costly. However, previous studies have shown that fine-tuning with a small number of samples can still yield high-accuracy gesture classification^26,38^. In our study, we trained the model on a minimal dataset of just two subjects and then used fine-tuning to generalize the model to a third subject. With minimal calibration, the system achieved high accuracy, suggesting that transfer learning is a viable approach for our model, enabling effective adaptation to new users with minimal data and effort.

Our model robustness testing indicates a high level of user safety in the event of unexpected sensor failure during operation. We evaluated extreme scenarios, including the complete failure of one or more sensors. Although model performance drops noticeably under these conditions, the accuracy remains sufficient for the system to capture the correct motion in real-time, albeit with some minor difficulties, such as needing to repeat movements until the model makes the correct inference. The performance drop could likely be mitigated further by augmenting the training data with examples of faulty channels. However, since it is impractical to anticipate every possible mode of sensor failure, we opted to use a zero-signal approach as a proof-of-concept example of an unencountered faulty signal. This demonstrates the model’s ability to handle sensor anomalies it was not explicitly trained on.

In conclusion, we present a robust system for accurate motion classification using surface EMG and IMU signals, with the highest accuracy achieved by a CNN that integrates both data modalities. The model shows strong generalizability to new users, requiring only minimal fine-tuning to maintain high performance. It also demonstrates resilience to individual sensor failures, continuing to deliver reliable accuracy even under suboptimal conditions. Furthermore, we validate the use of towel-based electrodes for motion intention applications, reinforcing the findings of Tang et al., who demonstrated accurate digit classification of quasistatic movements such as hand gesture recognition using this innovative electrode system^26^. To support further research and development, we have made our code for data collection and analysis, as well as our datasets, publicly available, enabling the development of similar systems and machine learning models across the field.

The present study has several limitations. First, we evaluated only a limited set of movements, sufficient for navigating barrier-free environments, but not encompassing more complex tasks such as stair ascent and descent. For comprehensive exoskeleton control, it will also be necessary to predict continuous joint kinematics in addition to discrete motion classes, which will require the collection of ground-truth joint angle data. Hybrid LSTM–CNN architectures have shown promise for this purpose and could be explored in future work^39^. Second, the dataset included only three subjects; although transfer learning experiments indicate good generalizability, larger and more diverse cohorts are needed to confirm robustness. A fourth IMU on the left foot would provide a more complete dataset, though in practice most exoskeleton systems instrument only a single leg, where our results already demonstrate high accuracy irrespective of additional sensors. Third, the current system requires the attachment of multiple EMG sensors, which can be time-consuming. This limitation could be mitigated by embedding textile electrodes into a wearable sleeve, simplifying setup and improving usability. Finally, our experiments were conducted in healthy subjects that were not wearing an exoskeleton. Transfer learning suggests strong potential for adaptation to patients with minimal additional calibration, but validation in clinical populations remains essential. Future work will focus on expanding the dataset, including joint angle measurements, and integrating the system into a functional exoskeleton to support safe, real-world use.

## Methods

### EMG sensor fabrication

The conventional wet gel electrodes from a commercial three-electrode gel EMG setup (SeeedStudio EMG Detector) were replaced with custom-made textile electrodes, utilizing a graphene/PEDOT:PSS composite material^26^. The electrodes were fabricated using commercial cotton towels (NU249W, Aston Pharma), which were first cleaned by treatment with ethanol and UV/ozone. The cleaned towels were then soaked in a graphene solution for 30 minutes, followed by drying at 120$^{\circ }$C for an additional 30 minutes. This process was repeated with an aqueous solution of PEDOT:PSS (ph1000, Ossila, diluted 1:10). The alternating cycles of soaking in graphene and PEDOT:PSS were repeated several times to create the final composite material.

### Data collection

The EMG detector boards were connected to an ESP32-S3 microcontroller development board, which transmitted the data wirelessly over WiFi to a laptop. To measure motion, open-source commercially-available EmotiBit sensor units including 9-axis IMUs were employed^40^. The firmware for the IMU microcontrollers was manually developed using the manufacturer’s software^41^ and the Espressif 32 functionality provided by PlatformIO. Data were wirelessly transmitted using the EmotiBit Oscilloscope Software (1.11.1)^42^, which streamed the data locally over a UDP stream. The dataset comprises five distinct classes of motion (Turn left (90$^{\circ }$), Turn right (90$^{\circ }$), Picking up an object from the floor, Walking forwards and Walking backwards), with each class containing approximately 100 samples per subject, ensuring equal distribution across the dataset. Accurate IMU-to-segment or global-frame alignment is important for many biomechanical applications^43,44^; however, in our context of supervised motion classification, precise anatomical alignment was not required. Instead, IMUs were positioned consistently on each limb, and a simple calibration was performed by verifying in quiet standing that the device’s vertical axis measured approximately 1 g. The resulting local-frame vertical acceleration channel was then used directly as a feature for classification.

### Human subject study

Ethical approval for the study was granted by the University of Cambridge Department of Engineering Research Ethics Committee (registration number #394). All data were collected anonymously in accordance with the principles of the Helsinki Declaration. All participants provided informed consent prior to the experiment. Data collection took place in the Human Performance Laboratory at the University of Cambridge. The study involved three young healthy volunteer participants (2 females, 1 male), with an average age of $23 \pm 1$ years, height $173 \pm 9$ cm, and weight $69 \pm 16$ kg. EMG data were collected bilaterally for tibialis anterior, gastrocnemius medial and lateral heads, and soleus muscles. The electrodes were placed according to the SENIAM guidelines^45^. The 3 IMU units were placed on the right and left shanks, and right foot. Informed consent for the publication of the images in an online open-access journal was obtained from the subject depicted in Fig. 1.

### Timestamp matching between modalities

The raw UDP stream from the EmotiBit oscilloscope was divided into three distinct files, each corresponding to data from a specific sensor. These files were processed using the EmotiBit DataParser, producing output files for the nine modalities associated with each IMU. The timestamps of the two recorded signals were then aligned to ensure synchronization. To match the frequency of the EMG signal, the IMU data were upsampled by a factor of 40 using linear interpolation. The final output for each trial was a CSV file, containing 35 columns and 5000 rows, representing a 5-second recording sampled at 1000 Hz.

### Signal pre-processing

**Fig. 5:**
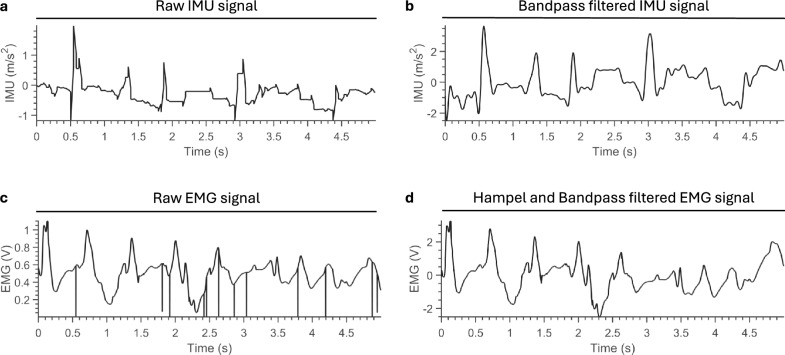
Signal processing. Representative examples of EMG and IMU signal processing recorded during a forward walking trial. (**a**) The raw IMU signals are bandpass filtered to reduce noise (**b**). (**c**) EMG signals undergo outlier removal using a Hampel filter before bandpass filtering (**d**).

Signal processing was performed in MATLAB (version R2023b). The raw EMG values were converted to voltage *V* using the equation

1 $$\begin{aligned} V=\frac{1.1}{4095} \cdot S \end{aligned}$$

where *S* are the raw signals and *V* is the output voltage in Volt, knowing that the on-board analog-to-digital converter (ADC) featured a 12-bit resolution and a reference voltage of 1.1 V. The signals were then passed through a Hampel filter that removed outliers deviating by more than three standard deviations from the 50 surrounding samples (see Fig. 5)^46^. The signals were then bandpass filtered using a fifth order Butterworth filter with cutoff frequencies of 0.2 and 400 Hz^47^. The signals were then normalized to zero mean and unit variance. Similarly, the raw IMU signals were bandpass filtered using a fifth-order Butterworth filter, but with cutoff frequencies of 0.2 Hz and 10 Hz, chosen based on the anticipated duration of the movements performed during the trial. These IMU signals were then normalized following the same procedure as the EMG signals.

### Machine learning models

All models were implemented and trained using the MATLAB Deep Learning Toolbox (version 23.2). The dataset, consisting of multichannel recordings with corresponding motion labels, was randomly split into training (80 %) and testing (20 %) sets for each run.

**Convolutional Neural Network (CNN).** The CNN architecture was adapted from Tang et al.^26^, with multiple one-dimensional convolutional blocks (convolution, batch normalization, ReLU activation, max pooling), followed by dropout, fully connected layers, and a softmax classification layer. A schematic is shown in Fig. 3 and model parameters are detailed in Table 3. **LSTM.** The LSTM model was implemented as a sequence-to-label classifier with an initial 1D convolution and max-pooling stage for temporal downsampling, followed by a recurrent head. Specifically, the network included a 100-unit LSTM layer, dropout, a fully connected layer, and a softmax output for classification into five motion classes. **Support Vector Machine (SVM).** As a classical machine learning baseline, we implemented a linear SVM. The model was trained using flattened feature vectors derived from the same pre-processed windows as the deep learning models. The choice of a linear kernel ensured scalability, as kernel-based SVMs are computationally prohibitive on large, high-dimensional datasets such as ours^48^. **Random classifier.** To establish a chance-level baseline, we implemented a random predictor that assigned class labels uniformly at random.

**Training procedure.** All neural models were trained with categorical cross-entropy loss and optimized with Adam (initial learning rate $1.5\times 10^{-3}$, batch size 50, 15epochs). Training was repeated with five random seeds, and results are reported as mean ± standard deviation of accuracy, precision, recall, and F1-score. Training was performed on a Dell XPS 13 laptop (Intel i7 CPU, 16 GB RAM), with individual CNN and LSTM training runs not exceeding five minutes.

**Transfer learning.** To assess generalization across users, we used a leave-one-subject-out protocol. Models were pre-trained on two subjects, then fine-tuned on 10 calibration samples per class from the held-out subject. Fine-tuning was performed with a reduced learning rate ($5\times 10^{-4}$) while freezing batch-normalization statistics, which improved stability under few-shot conditions. Three variants were evaluated: pre-trained-only (no calibration), fine-tuned (pre-trained + calibration), and FT-only (calibration without pre-training).

**Time-series classification with sliding windows.** To approximate continuous deployment, test data segments were concatenated into a long sequence and classified using a sliding window equal to the CNN input width. Predictions were obtained at strides of 1 s and 2 s. To improve temporal consistency, class probability scores were further smoothed using a per-class Kalman filter (process noise variance $Q = 2\times 10^{-3}$, measurement noise variance $R = 2\times 10^{-2}$). Accuracy was computed as the fraction of correctly predicted window centers relative to ground-truth motion labels.

**Table 3:** Parameter calculation for the updated CNN. Output shapes are shown as [batch size, channels, length]; layers that collapse the time dimension are noted accordingly.

Layer (type)	Output shape	Parameters
SequenceInput	[50, InputSize]	0
Conv1D-1 ($k{=}9$, $C_{out}{=}10$, same)	[50, 10, 5000]	3160
BatchNorm-1	[50, 10, 5000]	20
ReLU-1	[50, 10, 5000]	0
MaxPool1D (50, stride 50)	[50, 10, 100]	0
Conv1D-2 ($k{=}9$, $C_{out}{=}20$, same)	[50, 20, 100]	1820
BatchNorm-2	[50, 20, 100]	40
ReLU-2	[50, 20, 100]	0
MaxPool1D (10, stride 10)	[50, 20, 10]	0
Conv1D-3 ($k{=}9$, $C_{out}{=}30$, same)	[50, 30, 10]	5430
BatchNorm-3	[50, 30, 10]	60
ReLU-3	[50, 30, 10]	0
MaxPool1D (10, stride 10)	[50, 30, 1]	0
Conv1D-4 ($k{=}9$, $C_{out}{=}40$, same)	[50, 40, 1]	10840
BatchNorm-4	[50, 40, 1]	80
ReLU-4	[50, 40, 1]	0
GlobalAveragePooling1D	[50, 40]	0
Dropout ($p{=}0.5$)	[50, 40]	0
FullyConnected ($40{\rightarrow }5$)	[50, 5]	205
Softmax	[50, 5]	0
Classification	[50, 5]	0
**Total parameters**	–	**21,655**
**Trainable parameters**	–	**21,655**
**Non-trainable parameters**	–	**0**

### Statistical analysis

Model performance was evaluated using accuracy, precision, recall, and F1 scores, as outlined in Hicks et al.^49^. Accuracy, the most straightforward metric, represents the fraction of correctly classified samples and is calculated as:

2 $$\begin{aligned} \text {Accuracy} = \frac{TC}{TC + FC} \end{aligned}$$

where *TC* is the number of true classifications, and *FC* represents false classifications across all samples. This metric is particularly suitable for our evaluation because our dataset is balanced, ensuring that accuracy provides a meaningful overall assessment.

Precision measures how many of the samples predicted to belong to a particular class actually do, and is given by:

3 $$\begin{aligned} \text {Precision} = \frac{TP}{TP + FP} \end{aligned}$$

Here, *TP* represents true positives, and *FP* denotes false positives for a specific class. Precision is useful in cases where minimizing false positives is critical.

Recall, on the other hand, quantifies the proportion of actual class members that were correctly identified, and is defined as:

4 $$\begin{aligned} \text {Recall} = \frac{TP}{TP + FN} \end{aligned}$$

In this case, *FN* refers to false negatives, or instances where the model failed to identify the true class. Recall is valuable when minimizing false negatives is a priority.

Finally, the F1 score combines precision and recall into a single metric that balances both, particularly useful when there is an uneven trade-off between the two. The F1 score is calculated as:

5 $$\begin{aligned} \text {F1} = \frac{2 \cdot \text {Precision} \cdot \text {Recall}}{\text {Precision} + \text {Recall}} \end{aligned}$$

This harmonic mean provides a robust measure when dealing with imbalanced class distributions or when both precision and recall are equally important in evaluating model performance.

## Data Availability

The datasets generated and/or analysed during the current study are available in the Apollo repository and can be accessed via this link https://doi.org/10.17863/CAM.113504. The dataset can also be requested from Silas Ruhrberg Estévez (sr933@cam.ac.uk).

## Code availability

The underlying code for this study is available on GitHub and can be accessed via this link https://github.com/Sr933/exoskeleton. The code can also be requested from Silas Ruhrberg Estévez (sr933@cam.ac.uk).

## References

[1] Gu, D., Andreev, K. & E. Dupre, M. Major trends in population growth around the world. *China CDC Wkly.* **3**, 604–613, 10.46234/ccdcw2021.160 (2021). 10.46234/ccdcw2021.160 PMC8393076 34594946

[2] Mahlknecht, P. et al. Prevalence and burden of gait disorders in elderly men and women aged 60–97 years: A population-based study. *PLoS ONE* **8**, e69627. 10.1371/journal.pone.0069627 (2013). 23894511 10.1371/journal.pone.0069627 PMC3722115

[3] Florence, C. S. et al. Medical costs of fatal and nonfatal falls in older adults. *J. Am. Geriatr. Soc.* **66**, 693–698. 10.1111/jgs.15304 (2018). 29512120 10.1111/jgs.15304 PMC6089380

[4] The human cost of falls 2013; UK Health Security Agency — ukhsa.blog.gov.uk. https://ukhsa.blog.gov.uk/2014/07/17/the-human-cost-of-falls/. [Accessed 24-10-2024].

[5] Grimmer, M., Riener, R., Walsh, C. J. & Seyfarth, A. Mobility related physical and functional losses due to aging and disease - a motivation for lower limb exoskeletons. *J. NeuroEngineering Rehabil.* **16**, 10.1186/s12984-018-0458-8 (2019). 10.1186/s12984-018-0458-8 PMC6318939 30606194

[6] Raitor, M., Ruggles, S. W., Delp, S. L., Liu, C. K. & Collins, S. H. Lower-limb exoskeletons appeal to both clinicians and older adults, especially for fall prevention and joint pain reduction. *IEEE Transactions on Neural Syst. Rehabil. Eng.* **32**, 1577–1585. 10.1109/TNSRE.2024.3381979 (2024). 10.1109/TNSRE.2024.3381979 38536680

[7] Shi, B. et al. Wearable ankle robots in post-stroke rehabilitation of gait: A systematic review. *Front. Neurorobotics* **13**, 10.3389/fnbot.2019.00063 (2019). 10.3389/fnbot.2019.00063 PMC6700322 31456681

[8] Androwis, G. J. et al. A pilot randomized controlled trial of robotic exoskeleton-assisted exercise rehabilitation in multiple sclerosis. *Multiple Scler. Relat. Disord.* **51**, 10.1016/j.msard.2021.102936 (2021). 10.1016/j.msard.2021.102936 33878619

[9] Hunt, M. et al. Effectiveness of robotic exoskeletons for improving gait in children with cerebral palsy: A systematic review. *Gait & Posture* **98**, 343–354. 10.1016/j.gaitpost.2022.09.082 (2022). 36306544 10.1016/j.gaitpost.2022.09.082

[10] Cho, Y. K., Kim, K., Ma, S. & Ueda, J. A robotic wearable exoskeleton for construction worker’s safety and health. In *Construction Research Congress 2018*, vol. 2013, 19–28, 10.1061/9780784481288.003 (American Society of Civil Engineers, 2018).

[11] Yan, Z. et al. Development and testing of a wearable passive lower-limb support exoskeleton to support industrial workers. *Biocybern. Biomed. Eng.* **41**, 221–238. 10.1016/j.bbe.2020.12.010 (2021).

[12] Sado, F., Yap, H. J., Ghazilla, R. A. R. & Ahmad, N. Design and control of a wearable lower-body exoskeleton for squatting and walking assistance in manual handling works. *Mechatronics* **63**, 102272. 10.1016/j.mechatronics.2019.102272 (2019).

[13] Wang, Z. et al. A semi-active exoskeleton based on emgs reduces muscle fatigue when squatting. *Front. Neurorobotics ***15**, 10.3389/fnbot.2021.625479 (2021). 10.3389/fnbot.2021.625479 PMC8056132 33889081

[14] Wei, W., Zha, S., Xia, Y., Gu, J. & Lin, X. A hip active assisted exoskeleton that assists the semi-squat lifting. *Appl. Sci.* **10**, 2424. 10.3390/app10072424 (2020).

[15] Tiboni, M., Borboni, A., Vérité, F., Bregoli, C. & Amici, C. Sensors and actuation technologies in exoskeletons: A review. *Sensors* **22**, 884. 10.3390/s22030884 (2022). 35161629 10.3390/s22030884 PMC8839165

[16] Etenzi, E., Borzuola, R. & Grabowski, A. M. Passive-elastic knee-ankle exoskeleton reduces the metabolic cost of walking. *J. NeuroEngineering Rehabil. ***17**, 10.1186/s12984-020-00719-w (2020). 10.1186/s12984-020-00719-w PMC7385868 32718344

[17] Slade, P., Kochenderfer, M. J., Delp, S. L. & Collins, S. H. Personalizing exoskeleton assistance while walking in the real world. *Nature* **610**, 277–282. 10.1038/s41586-022-05191-1 (2022). 36224415 10.1038/s41586-022-05191-1 PMC9556303

[18] Chen, B. et al. A wearable exoskeleton suit for motion assistance to paralysed patients. *J. Orthop. Transl.* **11**, 7–18. 10.1016/j.jot.2017.02.007 (2017). 10.1016/j.jot.2017.02.007 PMC5866401 29662765

[19] Tang, C. et al. From brain to movement: Wearables-based motion intention prediction across the human nervous system. *Nano Energy* **115**, 108712. 10.1016/j.nanoen.2023.108712 (2023).

[20] Wang, D., Gu, X. & Yu, H. Sensors and algorithms for locomotion intention detection of lower limb exoskeletons. *Med. Eng. & Phys.* **113**, 10.1016/j.medengphy.2023.103960 (2023). 10.1016/j.medengphy.2023.103960 36966000

[21] Zhou, Y. User experience of lower extremity exoskeletons and its improvement methodologies: A narrative review. *Proc. Inst. Mech. Eng. Part H: J. Eng. Medicine* **238**, 1052–1068. 10.1177/09544119241291194 (2024). 10.1177/09544119241291194 39552186

[22] Cervantes, J., Garcia-Lamont, F., Rodríguez-Mazahua, L. & Lopez, A. A comprehensive survey on support vector machine classification: Applications, challenges and trends. *Neurocomputing* **408**, 189–215. 10.1016/j.neucom.2019.10.118 (2020).

[23] Li, M. et al. A cnn-lstm model for six human ankle movements classification on different loads. *Front. Hum. Neurosci.* **17**, 10.3389/fnhum.2023.1101938 (2023). 10.3389/fnhum.2023.1101938 PMC10030731 36968785

[24] Cheng, H.-R., Cao, G.-Z., Li, C.-H., Zhu, A. & Zhang, X. A cnn-lstm hybrid model for ankle joint motion recognition method based on semg. In *2020 17th International Conference on Ubiquitous Robots (UR)*, 339–344, 10.1109/UR49135.2020.9144698 (2020).

[25] Kim, J. et al. Soft wearable flexible bioelectronics integrated with an ankle-foot exoskeleton for estimation of metabolic costs and physical effort. *npj Flex. Electron.* **7**, 10.1038/s41528-023-00239-2 (2023).

[26] Tang, C., Yi, W., Kumar, S., Virk, G. S. & Occhipinti, L. G. Emg-based human motion analysis: A novel approach using towel electrodes and transfer learning. *IEEE Sensors Journal* **24**, 9115–9123. 10.1109/JSEN.2024.3354307 (2024).

[27] Lai, A. et al. In vivo behavior of the human soleus muscle with increasing walking and running speeds. *J. Appl. Physiol.* **118**, 1266–1275. 10.1152/japplphysiol.00128.2015 (2015). 25814636 10.1152/japplphysiol.00128.2015

[28] Kim, Y.-w., Kim, T.-h., Yang, M.-n., Yon, Y.-s. & Lee, J.-h. Comparison of activities of tibialis anterior, peroneus longus, and tibialis posterior muscles according to lunge squats and bulgarian split squats in a healthy population. *J. KEMA* **1**, 26–30, 10.29273/jkema.2017.1.1.26 (2017).

[29] Shi, B. et al. Wearable ankle robots in post-stroke rehabilitation of gait: A systematic review. *Front. Neurorobotics* **13**, 10.3389/fnbot.2019.00063 (2019). 10.3389/fnbot.2019.00063 PMC6700322 31456681

[30] Byrne, C. et al. Rectus femoris surface myoelectric signal cross-talk during static contractions. *J. Electromyogr. Kinesiol.* **15**, 564–575. 10.1016/j.jelekin.2005.03.002 (2005). 15946862 10.1016/j.jelekin.2005.03.002

[31] AL-Quraishi, M. S. et al. Classification of ankle joint movements based on surface electromyography signals for rehabilitation robot applications. *Med. & Biol. Eng. & Comput.* **55**, 747–758, 10.1007/s11517-016-1551-4 (2016). 10.1007/s11517-016-1551-4 27484411

[32] Bangaru, S. S., Wang, C. & Aghazadeh, F. Data quality and reliability assessment of wearable emg and imu sensor for construction activity recognition. *Sensors* **20**, 5264. 10.3390/s20185264 (2020). 32942606 10.3390/s20185264 PMC7570501

[33] Mengarelli, A. et al. Toward a minimal semg setup for knee and ankle kinematic estimation during gait. In *2023 IEEE 36th International Symposium on Computer-Based Medical Systems (CBMS)*, 293–298, 10.1109/cbms58004.2023.00233 (IEEE, 2023).

[34] Alzubaidi, L. et al. Review of deep learning: concepts, cnn architectures, challenges, applications, future directions. *J. Big Data* **8**, 10.1186/s40537-021-00444-8 (2021). 10.1186/s40537-021-00444-8 PMC8010506 33816053

[35] Si, X., Dai, Y. & Wang, J. Recognition of lower limb movements based on electromyography (emg) texture maps. In *2022 IEEE 5th International Conference on Electronics Technology (ICET)*, 1091–1095, 10.1109/ICET55676.2022.9824410 (2022).

[36] Nazmi, N. et al. A review of classification techniques of emg signals during isotonic and isometric contractions. *Sensors* **16**, 1304. 10.3390/s16081304 (2016). 27548165 10.3390/s16081304 PMC5017469

[37] Huang, C., Chen, M., Zhang, Y., Li, S. & Zhou, P. Model-based analysis of muscle strength and emg-force relation with respect to different patterns of motor unit loss. *Neural Plast.* **1–9**, 2021. 10.1155/2021/5513224 (2021). 10.1155/2021/5513224 PMC8245245 34257638

[38] Cote-Allard, U. et al. Deep learning for electromyographic hand gesture signal classification using transfer learning. *IEEE Transactions on Neural Syst. Rehabil. Eng.* **27**, 760–771. 10.1109/tnsre.2019.2896269 (2019). 10.1109/TNSRE.2019.2896269 30714928

[39] Zhu, M. et al. semg-based lower limb motion prediction using cnn-lstm with improved pca optimization algorithm. *J. Bionic Eng.* **20**, 612–627. 10.1007/s42235-022-00280-3 (2022).

[40] EmotiBit. EmotiBit.com — emotibit.com. https://www.emotibit.com/. [Accessed 26-08-2024].

[41] Releases · EmotiBit/ofxEmotiBit — github.com. https://github.com/EmotiBit/ofxEmotiBit/releases. [Accessed 24-10-2024].

[42] EmotiBit — github.com. https://github.com/EmotiBit. [Accessed 02-09-2025].

[43] Fan, B., Li, Q., Tan, T., Kang, P. & Shull, P. B. Effects of imu sensor-to-segment misalignment and orientation error on 3-d knee joint angle estimation. *IEEE Sensors J.* **22**, 2543–2552. 10.1109/JSEN.2021.3137305 (2022).

[44] Scattolini, M. et al. Inertial sensing for human motion analysis: Enabling sensor-to-body calibration through an anatomical and functional combined approach. *IEEE Transactions on Neural Syst. Rehabil. Eng.* **33**, 1853–1862. 10.1109/tnsre.2025.3567833 (2025). 10.1109/TNSRE.2025.3567833 40333092

[45] Recommendations for sensor locations in lower leg or foot muscles — seniam.org. http://seniam.org/lowerleg_location.htm. [Accessed 24-10-2024].

[46] Bhowmik, S., Jelfs, B., Arjunan, S. P. & Kumar, D. K. Outlier removal in facial surface electromyography through hampel filtering technique. In *2017 IEEE Life Sciences Conference (LSC)*, 258–261, 10.1109/LSC.2017.8268192 (2017).

[47] Movement artifact and baseline noise contamination. De Luca, C. J., Donald Gilmore, L., Kuznetsov, M. & Roy, S. H. Filtering the surface emg signal. *J. Biomech.* **43**, 1573–1579. 10.1016/j.jbiomech.2010.01.027 (2010). 20206934 10.1016/j.jbiomech.2010.01.027

[48] Ben-Hur, A. & Weston, J. *A User’s Guide to Support Vector Machines*, 223–239 (Humana Press, 2009). 10.1007/978-1-60327-241-4_13 20221922

[49] Hicks, S. A. et al. On evaluation metrics for medical applications of artificial intelligence. *Sci. Reports* **12**, 10.1038/s41598-022-09954-8 (2022). 10.1038/s41598-022-09954-8 PMC8993826 35395867

